# The Association between Preoperative Dry Eye Symptoms and Postoperative Discomfort in Patients Underwent Photorefractive Keratectomy

**DOI:** 10.1155/2019/7029858

**Published:** 2019-02-18

**Authors:** Gilad Rabina, Ingibjorg Iris Boguslavsky, Michael Mimouni, Igor Kaiserman

**Affiliations:** ^1^Department of Ophthalmology, Tel Aviv Sourasky Medical Center, Affiliated to the Sackler Faculty of Medicine, Tel Aviv University, Tel Aviv, Israel; ^2^Department of Ophthalmology, Barzilai University Medical Center, Ashkelon, Israel; ^3^Department of Ophthalmology, Rambam Health Care Campus, Affiliated to the Bruche and Ruth Rappaport Faculty of Medicine, Technion-Israel Institute of Technology, Haifa, Israel; ^4^Faculty of Health Science, Ben Gurion University, Be'er Sheva, Israel; ^5^Care Vision Laser Refractive Center, Tel Aviv, Israel

## Abstract

**Purpose:**

To investigate the association between preoperative dry eye symptoms on postoperative pain and discomfort after photorefractive keratectomy (PRK).

**Methods:**

A retrospective case series of 151 consecutive patients, who underwent myopic PRK in both eyes between 5/2016 and 5/2017. Patients with positive dry eye disease (DED) signs on clinical examination or with known DED were excluded. Patients underwent a subjective evaluation for dry eye symptoms using ocular surface disease index (OSDI) and modified standard patient evaluation of eye dryness (SPEED) questionnaires. One day postoperatively, the patients were evaluated again by a questionnaire of pain, discomfort, photophobia, foreign body sensation, satisfaction with vision, and frequency of usage of anesthetic drops.

**Results:**

Fifty-two patients had any preoperative dry eye symptoms (OSDI score > 0) compared to 99 nonsymptomatic patients (OSDI score of 0). Postoperatively, the symptomatic dry eye patients suffered significantly more pain than the nondry eye patients (*p*=0.02). Thirteen patients had a cumulated modified SPEED score >4 (moderate to severe) in comparison to 138 patients with score of 0–4 (non to mild). Patients with moderate to severe preoperative symptoms suffered more pain (*p*=0.006), photophobia (*p*=0.005), and epiphora (*p*=0.03). No statistically significant difference was seen in postoperative subjective visual quality (*p*=0.82) between the two groups.

**Conclusion:**

Preoperative dry eye symptoms may be associated with postoperative pain, epiphora, and photophobia and thus influence negatively on patient satisfaction with this procedure.

## 1. Introduction

Dry eye disease (DED) is very common ocular condition with a prevalence of 7.4% to 33.7% [1–5]. According to the National Eye Institute/Industry Dry Eye Workshop, DED is defined as “a disorder of the tear film due to tear deficiency or excessive evaporation, which causes damage to the interpalpebral ocular surface and associated with symptoms of ocular discomfort” [1]. DED can result from decreased tear production or increased tear evaporation. There are multiple known risk factors for DED, including low humidity, indoor environment, computer use, television watching, pollution, long-term contact lens wear, medications (such as antihistamines and antidepressants), and smoking [6–8]. The vicious cycle of DED includes tear film instability which leads to hyperosmolarity on the epithelial surface, which results in apoptosis of epithelial cells leading to inflammation and a loss of mucin-producing goblet cells. Eventually, the damage to the goblet cells becomes irreversible with permanent damage to the tear film [9, 10]. Chronic inflammation can also cause receptor and neurotransmitter changes, which results in pain modulation [11, 12]. These changes can amplify neural signals and increase the intensity of experienced pain. During photorefractive keratectomy (PRK), the corneal nerve endings that terminate in the anterior stroma and epithelium are disrupted. Thus, it is possible that a patient might have increased perception of pain following PRK. In addition, DED has been established as one of the most common postoperative complications of laser-assisted in situ keratomileusis (LASIK) and PRK and has been widely investigated [13–16]. The postoperative dry eye is usually transient but may influence negatively on the patient's satisfaction with procedure [17, 18].

The aim of this study is to investigate the effect of preoperative dry eye symptoms on postoperative pain and discomfort during the short-term recovery period after PRK.

## 2. Methods

All data for the study were collected and analyzed in accordance with the policies and procedures of the Institutional Review Board (IRB) of the Barzilai Medical Center and the tenets set forth in the declaration of Helsinki (approval number BRZ-0067-13).

### 2.1. Study Participants

This retrospective case series included consecutive patients, who underwent myopic PRK in both eyes between 5/2016 and 5/2017 at Care-Vision Laser Centers, Tel-Aviv, Israel. All patients were operated by a single surgeon (IK). Patients under the age of 18 and with history of known DED, previous ocular surgery, suspected keratoconus or keratoconus, active ocular disease, significant refractive media alterations, history of autoimmune disease, collagen disease, and diabetes and pregnant women were excluded from this study.

### 2.2. Preoperative Assessment

Preoperatively, each patient underwent complete ocular examination including Schirmer's test [19] and fluorescein tear breakup time (TBUT) [20]. Patients with Schirmer's score less than 10 mm/5 minutes or TBUT under 10 seconds were excluded. Patients underwent a subjective evaluation for DED by the valid and reliable ocular surface disease index (OSDI) [21] and modified standard patient evaluation of eye dryness (SPEED) [22] questionnaires. The modified SPEED questionnaire included questions regarding the severity of dry eye, itching, epiphora, ocular discharge, burning sensation, eye fatigue, sensation of a blurry screen in front of eyes, and ocular redness during the last month before the surgery. The score for each question ranges between 0 and 4 (0 = never, 1 = sometimes, 2 = half of the time, 3 = most of the time, and 4 = all the time).

### 2.3. Surgical Protocol

The epitehlium was removed with application of 20% alcohol for 20 seconds. PRK was performed with the EX500 laser with an optic zone of 6.5–7 mm (Wave Light Alcon, Erlanger, Germany). Mitomycin C was applied for 20 to 60 seconds according to refraction correction. A soft contact lens was inserted for 4-5 days until full epithelialization.

### 2.4. Patients Follow-Up

Patients were treated with topical moxifloxacin 0.5% (VIGAMOX) for a week and dexamethasone 0.1% (STERODEX) for a total of one month with gradual tapering down. Patients were provided with one ml of Benoxinate 0.15% and instructed to use in case of unbearable pain during the first 48 hours only. In addition, they were instructed to use oral acetaminophen in case of pain. Artificial tears were given as needed. One day postoperatively, patients were asked to answer a standard “post-op questionnaire.” The questionnaire included grade of pain, photophobia, foreign body sensation, satisfaction with vision, and frequency of usage of anesthetic drops. All questions were with a scale of 1–4 (1 = none, 2 = mild, 3 = moderate, and 4 = severe).

### 2.5. Dry Eye Questionnaires

Patients were divided into two groups according to their OSDI score. The first group had no preoperative DED symptoms (score = 0), and the second group of patients had any preoperative DED symptoms (score > 0). Patients were also divided into two groups according to their modified SPEED score. The first group had non to mild symptoms (score of 0–4), and the second group had moderate to severe symptoms (score > 4).

### 2.6. Sample Size Calculation

An internal pilot analysis (*n*=15) revealed that for patients without DED symptoms, the mean postoperative pain was 3.7 ± 1.5 and that there was a 2 : 1 ratio of patients without and with DED symptoms and a 20% increase in postoperative pain in patients with DED symptoms. Therefore, with an alpha of 0.05 and a power of 0.80, we calculated that ∼48 patients would be required in the OSDI > 0 group and ∼97 in the OSDI = 0 group.

### 2.7. Statistical Analysis

Data were entered in a spreadsheet (Excel 15.2, Microsoft Corp.) and analyzed with the SPSS statistical software version 23.0 (SPSS, Cary, NC, USA). For the analysis of continuous data, Student's *t*-test was used for normally distributed variables and Kruskal–Wallis for nonparametric variables. A 2-sided *p* value < 0.05 was considered statistically significant. Mean values are presented together with their standard deviations and medians with their ranges.

## 3. Results

A total of 151 consecutive patients, with a mean age of 24 ± 6 years, were included in this study. Contact lenses were preoperatively worn by 50.7% of them.

### 3.1. Preoperative OSDI Score and Post-PRK Pain and Discomfort

Fifty-two patients had any preoperative dry eye symptoms (OSDI score > 0) compared to 99 nonsymptomatic patients (OSDI score of 0). Symptomatic patients suffered significantly more pain in the first postoperative day than the nonsymptomatic patients (*p*=0.02). The other postoperative symptoms including photophobia, quality of vision, foreign body sensation, epiphora, and number of times of the anesthetic drops were used were not significantly different between the groups (Table 1).

### 3.2. Preoperative Refractive Error and Pain

**Figure 1:**
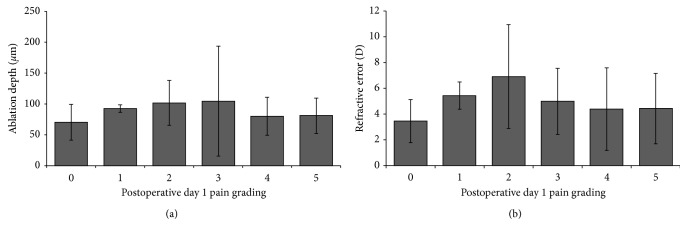
(a) Relationship between max ablation depth and pain on the first day post photorefractive keratectomy (PRK). (b) Relationship between refractive error and pain on the first day post photorefractive keratectomy (PRK).

There was a nonsignificant negative correlation between ablation depth (*μ*m) and postoperative day 1 pain (*r*=−0.15, *p*=0.08) and a significant negative correlation between refractive error and postoperative day 1 pain (*r*=−0.19, *p*=0.02). There was no significant difference in mean max ablation depth (*μ*m) between eyes with various pain grading (Figure 1(a)) with no meaningful trend in terms of refractive error (Figure 1(b)), the only significant difference being that patients with a pain of 5 had significantly lower refractive error than those with a pain of 2 (*p*=0.04).

### 3.3. Preoperative Contact Lens Wearing and Pain

There was a similar proportion of contact lens wearers in the OSDI > 0 group (52.9%) and the OSDI = 0 group (49.5%) (*p*=0.69). In addition, postoperative day 1 pain between contact lens wearers (3.67 ± 1.36) and those that did not wear contact lenses (3.71 ± 1.23) before the surgery was similar (*p*=0.86).

### 3.4. Preoperative Modified SPEED and Post-PRK Pain and Discomfort

**Figure 2:**
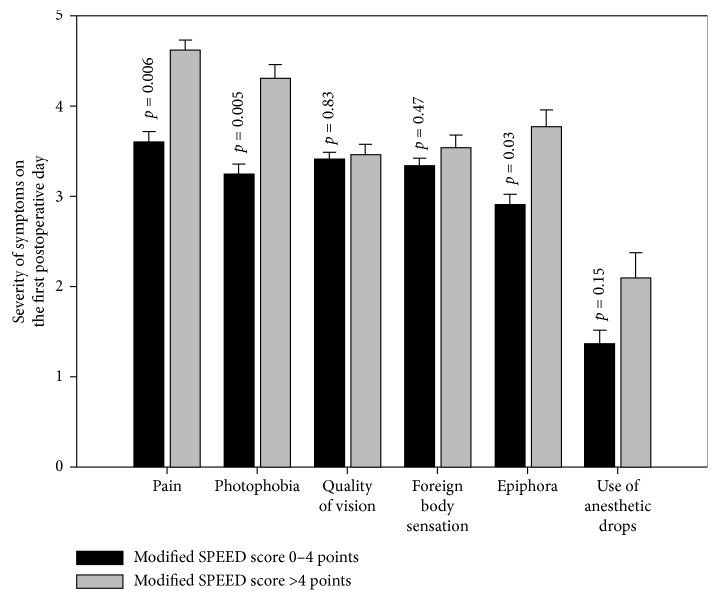
Relationship between preoperative positive modified SPEED questionnaire to discomfort on the first day post photorefractive keratectomy (PRK).

Thirteen patients had a cumulated modified SPEED score >4 (moderate to severe) in comparison to 138 patients with score of 0–4 (non to mild). Patients with moderate to severe preoperative symptoms suffered more pain (*p*=0.006), more photophobia (*p*=0.005), and more epiphora (*p*=0.03) in the first postoperative day than those with non to mild preoperative symptoms. No statistically significant difference was seen in postoperative quality of vision (*p*=0.82) or foreign body sensation (*p*=0.47) between the two groups in the first postoperative day (Figure 2).

### 3.5. Subanalysis of Modified SPEED Questionnaire

**Figure 3:**
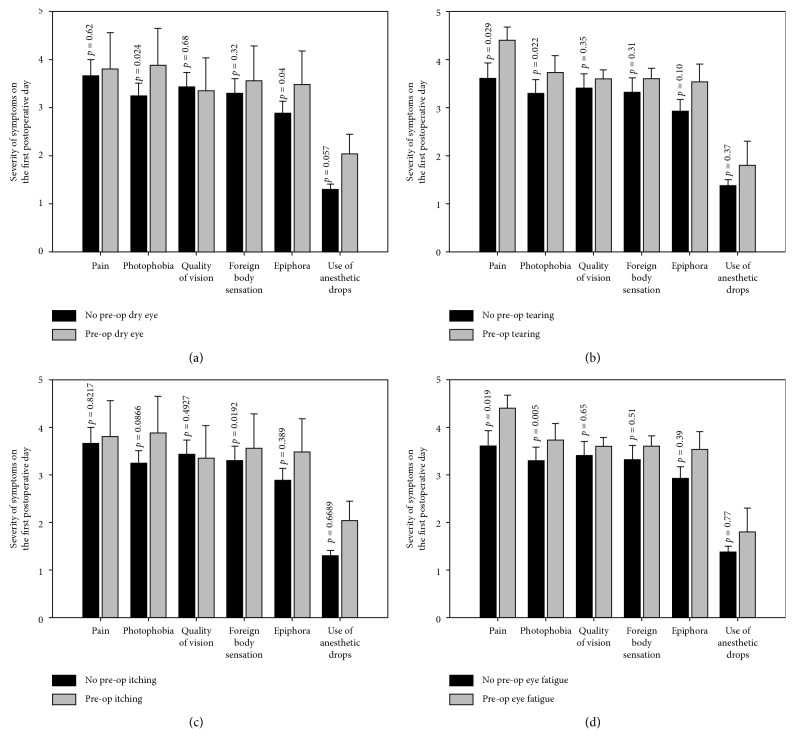
Relationship between preoperative dry eye symptoms to postoperative discomfort on the first day post photorefractive keratectomy (PRK). (a) Relationship between preoperative symptom of dry eye and postoperative discomfort. (b) Relationship between preoperative tearing and postoperative discomfort. (c) Relationship between preoperative eye itching and postoperative discomfort. (d) Relationship between preoperative eye fatigue and postoperative discomfort.

For each individual question of the modified SPEED questionnaire, an association was found with postoperative discomfort.  Figure 3 demonstrates patients that reported having preoperative dry eye symptoms (*n*=25) (Figure 3(a)) were significantly more likely to suffer from photophobia (*p*=0.024) and tearing (*p*=0.046) and to use more the anesthetic drops (*p*=0.047) in the first postoperative day. Patients that reported suffering from preoperative tearing (*n*=15) had statistically significant (*p*=0.02) more pain on the first postoperative day (Figure 3(b)). Patients that reported preoperative eye itching (*n*=30) suffered more from postoperative foreign body sensation (*p*=0.019) than those that did not (Figure 3(c)). Those reported having preoperative ocular burning sensation were more likely to suffer from postoperative pain (*p*=0.031) than those that did not. Patients reporting preoperative eye fatigue (*n*=33) were more likely to suffer from pain (*p*=0.019) and photophobia (*p*=0.005) the first day after the PRK and those reporting preoperative ocular redness (*n*=11) suffered significantly more from tearing (*p*=0.039) (Figure 3(d)).

## 4. Discussion

In this paper, the relationship between having preoperative any dry eye symptoms and post PRK pain, discomfort, epiphora, and quality of vision was examined. It was found that preoperative dry eye symptoms, assessed by the OSDI and modified SPEED questionnaires, without any objective findings, can significantly influence on the first postoperative day discomfort following PRK.

To understand how PRK in the presence of DED can accentuate pain, it is important to consider the innervation of the cornea and the perception of pain from the ocular surface. The cornea is mostly innervated by polymodal nociceptors, which are activated by chemical mediators upon corneal injury, heat, and cold. The main neurotransmitters are calcitonin gene related peptide and substance P, that also act as proinflammatory substances, promoting the release of other mediators resulting in “neurogenic inflammation.” Inflammatory mediators from the injured cells induce changes in the ion channel functioning by interacting with the nociceptor ending membrane, resulting in depolarization that increases the nerves excitability. This could explain the increased pain sensation in inflamed tissues. PRK damages the corneal nerve endings that terminate in the anterior stroma and epithelium. This damage may form increased sensory influx to second- and higher-order nociceptive neurons, which result in enhancement of their excitability, hyperalgesia, and allodynia [11, 23]. Chronic preoperative DED may contributed to the damage caused by PRK and exacerbate symptoms and findings on ocular examination.

Previous studies demonstrated that DED, resulted in decreased corneal sensation, decreased tear secretion, and tear film stability, after PRK [15, 16]. In addition, few studies found that preoperative findings of positive Schirmer's test, TBUT, corneal esthesiometry, and rose bengal staining can exacerbate DED follow PRK and were found as predictors for chronic dry eye follow PRK [15, 16, 24]. Bower et al. examined 143 patients and evaluated the manifestations of DED following PRK and LASIK. In their study, they found that patients who reported postoperative symptoms of dry eye had more clinical findings baseline up to twelve months postoperatively despite not having prior history or clinical findings of DED. They also found that 5% of eyes without a history of DED developed chronic DED after PRK and conclude that preoperatively lower Schirmer and TBUT scores tend to develop chronic dry eye after PRK, but the majority of patients would only experience it transiently and will have full recovery [15]. Similar findings were also found by Ang et al. and Quinto et al. [16, 24].

In meibomian gland dysfunction (MGD), the viscosity of the meibum is increased, possibly due to changes in meibum composition [25]. Stasis of meibum promotes growth of bacteria, releasing esterase and lipase. With increased enzyme activity, the viscosity and melting temperature of meibum is increased, leading to additional stasis, generating free fatty acids in turn causing chronic inflammation and hyperkeratinisation [26, 27].

Mild DED can result in dry eye symptoms, reflected in a positive OSDI or modified SPEED questionnaires without objective findings such as TBUT and Schirmer's test. Thus, it is sometimes difficult to clinically assess the severity of preoperative DED. In this study, we demonstrated that patients without preoperative clinical findings of DED and without positive dry eye symptoms complains will suffer less postoperative pain, epiphora, and photophobia. On the contrary, patients with mild preoperative dry eye symptoms, even without any objective clinical findings, may suffer more after PRK.

Interestingly, in the current study, there was no correlation between ablation depth and postoperative pain. In addition, there was a negative correlation between refractive error and postoperative pain. This contradicts the findings of Garcia et al. [28] who reported that patients with a −3 D to −5 D demonstrated greater early postoperative pain compared to those with lower refractive errors (−1 D to −3 D). It is worth mentioning that in their study, there was no association between refractive error and pain when using the multidimensional pain questionnaire, and such an association was only detected with the brief pain inventory questionnaire. They explained that perhaps some aspects of pain were better assessed with the latter.

The main limitations of this study are its retrospective nature and it being subjective as both preoperative dry eye symptoms and postoperative complains were evaluated by questionnaires. An additional limitation is the use of a nonvalidated post PRK pain questionnaire. Also, tear osmolality test and confocal microscopy are not performed routinely in our institution and were not examined in this current study. All patients in this study got both eyes operated at the same day, which may influence pain and discomfort. Last, due to the retrospective nature of this study, we cannot comment on the number of hours passed and the evaluation of pain; patients may have been examined anywhere between 14 hours and 36 hours following surgery in the postoperative day 1 visit.

## 5. Conclusions

Preoperative dry eye symptoms may influence pain, epiphora, and photophobia after PRK and thus influence negatively on patient satisfaction from this procedure. Preoperative dry eye and blepharitis treatment is recommended for patients with any dry eye symptoms, even without objective findings, prior to PRK.

## Figures and Tables

**Table 1:** Preoperative dry eye symptoms (OSDI score) and postoperative discomfort.

Parameter	OSDI = 0 (*n*=99)	OSDI > 0 (*n*=52)	*p* value
Preoperative contact lens (%)	49.5	52.9	0.69
Pain sensation	3.51	4.04	0.02
Photophobia	3.21	3.58	0.14
Quality of vision	3.37	3.50	0.33
Foreign body sensation	3.27	3.50	0.24
Tearing	3.03	2.88	0.52
Use of anesthetic drops	1.37	1.52	0.61

## Data Availability

The data used to support the findings of this study are available from the corresponding author upon request.
