# Erratum to “The Association between Preoperative Dry Eye Symptoms and Postoperative Discomfort in Patients Underwent Photorefractive Keratectomy”

**DOI:** 10.1155/2019/2650760

**Published:** 2019-06-16

**Authors:** Gilad Rabina, Ingibjorg Iris Boguslavsky, Michael Mimouni, Igor Kaiserman

**Affiliations:** ^1^Department of Ophthalmology, Tel Aviv Sourasky Medical Center, The Sackler Faculty of Medicine, Tel Aviv University, Tel Aviv, Israel; ^2^Department of Ophthalmology, Barzilai University Medical Center, Ashkelon, Israel; ^3^Department of Ophthalmology, Rambam Health Care Campus, The Bruche and Ruth Rappaport Faculty of Medicine, Technion-Israel Institute of Technology, Haifa, Israel; ^4^Faculty of Health Science, Ben Gurion University, Be'er Sheva, Israel; ^5^Care Vision Laser Refractive Center, Tel Aviv, Israel

In the article titled “The Association between Preoperative Dry Eye Symptoms and Postoperative Discomfort in Patients Underwent Photorefractive Keratectomy” [1], Dr. Shmuel Levartovsky was incorrectly included as an author. The corrected authors' list and affiliations are shown above and updated in place.
